# Association Between HIV-Related Tweets and HIV Incidence in the United States: Infodemiology Study

**DOI:** 10.2196/17196

**Published:** 2020-06-24

**Authors:** Robin Stevens, Stephen Bonett, Jacqueline Bannon, Deepti Chittamuru, Barry Slaff, Safa K Browne, Sarah Huang, José A Bauermeister

**Affiliations:** 1 Department of Family and Community Health University of Pennsylvania School of Nursing Philadelphia, PA United States; 2 University of California Merced Merced, CA United States; 3 University of Pennsylvania Philadelphia, PA United States; 4 Children's Hospital of Pennsylvania Philadelphia, PA United States

**Keywords:** HIV/AIDS, social media, youth, natural language processing, surveillance

## Abstract

**Background:**

Adolescents and young adults in the age range of 13-24 years are at the highest risk of developing HIV infections. As social media platforms are extremely popular among youths, researchers can utilize these platforms to curb the HIV epidemic by investigating the associations between the discourses on HIV infections and the epidemiological data of HIV infections.

**Objective:**

The goal of this study was to examine how Twitter activity among young men is related to the incidence of HIV infection in the population.

**Methods:**

We used integrated human-computer techniques to characterize the HIV-related tweets by male adolescents and young male adults (age range: 13-24 years). We identified tweets related to HIV risk and prevention by using natural language processing (NLP). Our NLP algorithm identified 89.1% (2243/2517) relevant tweets, which were manually coded by expert coders. We coded 1577 HIV-prevention tweets and 17.5% (940/5372) of general sex-related tweets (including emojis, gifs, and images), and we achieved reliability with intraclass correlation at 0.80 or higher on key constructs. Bivariate and multivariate analyses were performed to identify the spatial patterns in posting HIV-related tweets as well as the relationships between the tweets and local HIV infection rates.

**Results:**

We analyzed 2517 tweets that were identified as relevant to HIV risk and prevention tags; these tweets were geolocated in 109 counties throughout the United States. After adjusting for region, HIV prevalence, and social disadvantage index, our findings indicated that every 100-tweet increase in HIV-specific tweets per capita from noninstitutional accounts was associated with a multiplicative effect of 0.97 (95% CI [0.94-1.00]; *P*=.04) on the incidence of HIV infections in the following year in a given county.

**Conclusions:**

Twitter may serve as a proxy of public behavior related to HIV infections, and the association between the number of HIV-related tweets and HIV infection rates further supports the use of social media for HIV disease prevention.

## Introduction

The highest burden of new HIV infections has been reported in adolescents and young adults between the ages of 13 and 24 years, with 37.1% of the new HIV infections occurring in this age group in the United States [1]. Among the youths in this age group, 87% of the individuals diagnosed with HIV infection were reported to be young men, and 51% of these young men were identified as African American, while 25% of these young men were identified as Hispanic/Latino [1]. With the rapid increase in the usage of social media over the last 15 years, Twitter has emerged as a popular social networking platform. Studies have shown that Twitter is used by 32% of the adolescents and 44% of the young adults, with Black youths reporting higher levels of use than their white and Latino peers [2,3]. Since Twitter is used to discuss health-related and risk-related topics [4-6], this platform offers a distinct opportunity to investigate the attitudes, beliefs, and behaviors of the youths via their publicly shared posts that they have created or to which they have responded. This unique content may provide additional insights into the sentiments and discourses of youths [7] beyond what can be identified in traditional formative research methods, particularly at the national level. Analysis of Twitter, for example, might offer insight into the HIV-related beliefs and attitudes of youths of different races/ethnicities and help inform interventions that are designed to curb the HIV epidemic among youths.

The popularity of Twitter and the high volume of public tweets provide unprecedented access to discourses about sexual health and HIV by youths across a country. Although youths use social media platforms such as Twitter to share and seek sexual health information and to communicate with romantic and sexual partners [8], research on tweets related to alcohol, marijuana, cancer, and vaccines has shown that Twitter is also used to promote risky behaviors, spread misinformation, and reinforce HIV- and sexually transmitted infection (STI)-related stigmas [4,5,9,10]. Several studies have also considered social media messages as surveillance data to monitor the incidence of influenza, depression, Zika virus infections, and substance use [11-13]. Similar techniques have been used to assess the associations between social media messages on sex and HIV and the risk behavior and HIV incidence [14].

Several studies have shown evidence of a correlation between HIV-related tweets and HIV prevalence in a population [8,14,15]. Two studies [14,15] showed that future-oriented and action-based tweets regarding HIV were associated with decreased incidence of HIV infections at the county level in the United States. In contrast, Young et al [8] found that there was a statistically significant positive association between HIV-related tweets and HIV prevalence. However, these studies [8,14,15] did not distinguish between the source of the tweet; instead, they combined tweets from individual users and institutions such as public health agencies in their analyses. Importantly, studies on HIV and social media focus on certain keywords such as “sex,” “HIV testing,” and “discrimination.” Although the use of these keywords is useful for examining the associations between HIV-related tweets and HIV prevalence, studies often have reduced sensitivity to retrieve relevant tweets for analysis and intervention [16]. Since the abovementioned studies have provided promising evidence that tweets may be associated with HIV risk, there is a need for in-depth contextualized analysis of Twitter messages on risky sexual behavior and health, including analyses of message source variations. Therefore, the goal of our study was to explore how Twitter activity is related to HIV incidence and whether message characteristics such as content and source can reveal the incidence of HIV infection in a population and the future risks associated with HIV. Twitter messages may serve as a signal of the real-time dynamics in HIV epidemiology. In this study, we combined in-depth content analysis of HIV-related tweets with automated machine learning techniques to analyze the county-level associations between HIV-related tweets and new HIV infections in the United States.

## Methods

### Sample

**Figure 1:**
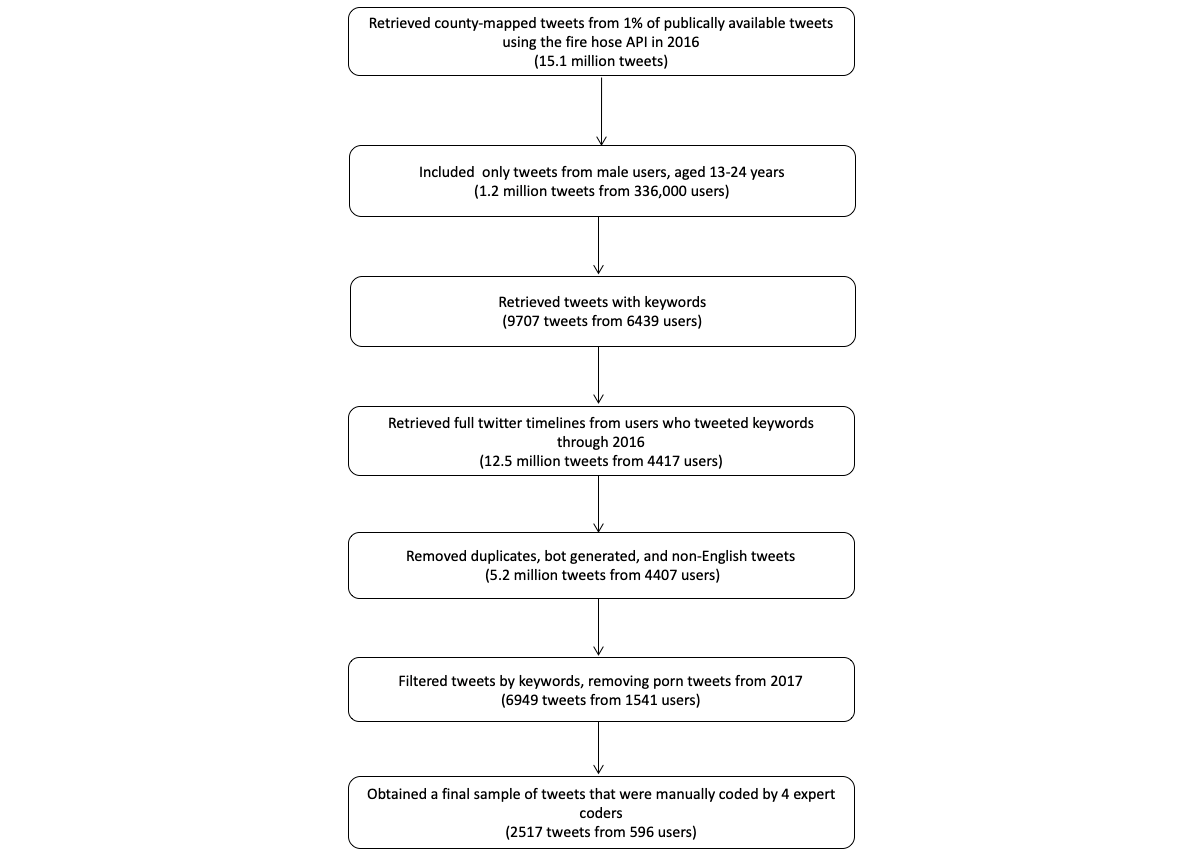
Twitter sample retrieval flowchart. API: application programming interface.

Using the Twitter “fire hose” application programming interface, which provides broad access to public Twitter data, we drew a random sample of 1% of publicly available tweets posted between January 1, 2016 and December 31, 2016. We sampled tweets from users who tweeted at least 500 words in 2016 and who were geolocated in a county in the United States. To determine the geolocation, we used two types of data: tweet-specific latitude/longitude coordinates and the self-reported location information in Twitter user profiles. The distribution of the geolocated tweets by county approximate the US population density [17]. Duplicate tweets, bots, and non-English tweets were removed [18,19]. After we produced age and gender affiliation estimates for each user with HIV-related tweets, based on our tested algorithms [20,21], we limited our sample to users with predicted age (range, 13-24 years) and predicted gender (males only). Using previous literature [22] and input from our young researchers, we developed a keyword list of HIV-related terms such as HIV, AIDS, HIV testing, condoms, multiple sexual partners, STI, risky sexual behavior, and pre-exposure prophylaxis (PrEP). PrEP is an effective HIV prevention medication taken prior to exposure to the virus. This keyword list was used to identify relevant tweets, and we extracted 9707 HIV-related tweets from 6439 users from the age/gender stratified sample. We also removed pornographic tweets by developing a classifier to identify pornography and excluded those tweets from our data set. Our final data set included 6949 tweets by 1541 young male adults and male adolescents in the United States, and these tweets contained at least one relevant keyword. Figure 1 shows the number of messages and users retained at each step of the above described process.

### Content Analysis

Data analysis was conducted with a sample of HIV-related tweets posted on Twitter. We manually coded a sample of HIV-related tweets by oversampling tweets with HIV-specific keywords. To accomplish this, we grouped our keywords into 2 broad categories: HIV prevention-specific tweets (n=1577) and general sex-related tweets (n=5372). We initially included a third category, namely, risk-related, which included risk behavior-promoting (n=6) tweets. However, we excluded this category from our analysis owing to the small number of tweets in this category*.* From the final data set, we took the full sample of 1577 prevention-related and a 17.5% (940/5372) random sample of general sex-related tweets, yielding 940 general sex-related tweets for manual content analysis.

The final data set (2517 tweets by 596 users) was coded by 4 expert coders for 19 nonexclusive categories. To capture the context, we expanded the coding unit beyond the initial tweet. The coders read the 5 tweets that proceeded from the tweet and the 5 tweets that followed the tweet. They also reviewed the images or webpages linked to the tweet. The coders achieved reliability by using a separate training data set of tweets, which was created through the same procedure used for the sample data set. We used a training set to train the coders without depleting the main data set. During training, the coders reconciled the differences in the code interpretations and coding approaches as a team. After the coding schema was finalized, the 4 coders achieved intercoder reliability on key constructs assessed with an intraclass correlation at 0.80 or higher. Approximately 20% (500/2517) of the final data set was coded by at least two coders.

### Measures

The HIV incidence—the outcome variable—was assessed as the number of new cases of HIV infections in a given county in 2017. These data were sourced from the Centers for Disease Control and Prevention AtlasPlus data platform [23]. Counties with suppressed data owing to low case counts were assigned a value of 2, which represented the midpoint between the lowest possible suppressed value of 1 and the highest possible suppressed value of 4.

Twitter messages were classified into the following 3 categories in 2016: risk-specific Twitter activity, prevention-specific Twitter activity, and HIV-specific Twitter activity. Risk-specific Twitter activity is the sum of all the tweets categorized with a risk-related code (eg, multiple partners, pro risk-taking, substance use, transactional sex, and unprotected sex) in a given county, per 100,000 residents. Prevention-specific Twitter activity is the sum of all the tweets categorized as prevention-related (eg, antirisk taking, condoms, HIV testing, HIV/AIDS, PrEP, research, education, and news) in a given county, per 100,000 residents. HIV-specific Twitter activity is the sum of the risk-specific and prevention-specific Twitter activities, in addition to the tweets that were tagged as related to LGBTQ content. All users in our data set were identified as either an individual or an institution based on the manual review of the user profile and recent posting activity. Institutions included public health agencies, social service organizations, and advocacy groups, and typically included the organization name in the username or user description. Our final measures of the tweets consisted of 3 Twitter activity categories (risk, prevention, or HIV-specific) from individuals or institutions, resulting in 6 Twitter variables.

We accounted for 3 geographic control variables: HIV prevalence, social disadvantage, and census region. HIV prevalence in a geographic area is the key epidemiological factor linked to the number of new cases in that area [24]. We used county-level HIV prevalence rates in 2015 to account for the existing patterns of HIV infection. Counties with suppressed data owing to low case counts were assigned case counts of 6, which represented the midpoint between the lowest possible suppressed value of 1 and the highest possible suppressed value of 11. Studies have also shown that socioeconomic factors measured at the city-wide level are the key drivers of new HIV infections [25]. To capture the combined effect of multiple dimensions of socioeconomic disadvantage, we calculated the social disadvantage index at the county level for the counties in our study (Cronbach α=.82) [26]. This index was calculated by summing the z-scores for the percentage of the population living in poverty, the percentage of the population with a high school degree or equivalent, the median household income, and the percentage of the population lacking health insurance. These measures were obtained from the US Census Bureau Small Area Income and Poverty Estimates and the American Community Survey [27]. Negative weights were applied to high school education and median income, yielding an index that reflected greater social disadvantage for high values of the index and lesser social disadvantage for low values of the index. Census region was included to account for the regional variations in the HIV epidemic. The four regions, that is, northeast, south, Midwest, and west regions of the United States, were treated as the control variables in the models.

### Statistical Analysis

General sex-related tweets were given sample weights of 6.25 for all the analyses to reflect the random samplings performed to reduce the data for coding. We used the Wilcoxon rank sum test with continuity correction for large samples to compare county tweet outputs based on the message source (ie, tweets from individuals vs institutions). We used negative binomial regression to estimate the effects of Twitter activity on HIV incidence at the county level. Our outcome of interest for this analysis was the rate of new diagnoses of HIV infections per capita at the county level. To model this rate variable, we included an offset term for the county population in 2017 in our regression analysis [28]. Negative binomial regression was chosen because our county outcome variables showed significant overdispersion from the Poisson distribution. Unadjusted models were run first for each of the 6 Twitter variables and the 3 control variables. Separate multivariate models were run for each of the Twitter variables, thereby adjusting for all the control variables. Variance inflation factors were examined for all final models, and none showed evidence of multicollinearity. Analyses were performed in R-3.5.1 [29] using the MASS package [30] glm.nb() function for negative binomial regression.

## Results

### Descriptive and Geospatial Data

Our data included 2517 tweets that were identified as potentially relevant to HIV risk (eg, unprotected sex) and prevention tags (eg, condom use, HIV testing, research, education), and these tweets originated in 109 counties across the United States. Of these, 940 were general sex-related tweets (including emojis, gifs, and images) and were given a sample weight to reflect our random sampling procedure. Each tweet in our data set represents 100 tweets in the real world as our data was drawn from 1% of publicly available tweets. However, we have reported all our results in units of true tweets, which were calculated by multiplying our results by 100. In 2016, 321 HIV-specific tweets, on an average, originated from individuals in each county. Counties had an average of 143 prevention-related and 118 risk-related tweets from individuals. An average of 944 HIV-specific tweets, 843 prevention-related tweets, and 31 risk-related tweets originating from institutions were sourced to each county. Institutions tweeted significantly more HIV-related (U=67,812; *P<*.001) and prevention-related messages (U=62,711; *P<*.001) and significantly less risk-related messages as compared to individuals (U=63,879; *P<*.001). Within counties that had at least one potentially relevant tweet, the median number of new HIV cases diagnosed in 2017 was 70 per county (range: 0-1530). HIV prevalence in these counties ranged from 6.02 to 2590 per 100,000 residents, with a median prevalence rate of 306 per 100,000 residents. The social disadvantage index ranged from –6.52 to 7.53 (Table 1).

The crude incidence rate ratios (IRRs) for each variable of interest of HIV incidence in 2017 were calculated using negative binomial regression with an offset for the county population (Table 2).

HIV prevalence in 2017 was positively associated with HIV prevalence in 2015 and social disadvantage index in 2015 (IRR 1.104, 95% CI 1.075-1.134; *P<*.001). Compared to that in the Midwest region, significantly higher HIV incidence was observed in the northeast (IRR 1.286, 95% CI 0.985-1.683; *P<*.001) and south (IRR 2.126, 95% CI 1.711-2.630; *P<*.001) regions of the United States. We did not observe a significant difference (IRR 0.967, 95% CI 0.749-1.250; *P>*.99) in the counties in the west region of the United States. The large number of prevention tweets from individuals in 2016 was significantly associated with the high incidence of HIV in the following year (IRR 1.082, 95% CI 1.003-1.183; *P*=.048). No other significant bivariate associations were found between HIV-related tweets and HIV incidence for combinations of tweet category and user type.

**Table 1:** Descriptive statistics at the county level (n=109).

	Values	
Descriptive statistics	Mean (SD)	Median (Min, Max)
HIV prevalence case count, 2017	173 (260)	70 (0.00, 1530)
HIV prevalence case count, 2015	484 (500)	306 (6.02, 2590)
County population, 2017	832,000 (1,090,000)	535,000 (13,900, 8,580,000)
Social disadvantage index, 2015	0.251 (3.06)	0.463 (–6.52, 7.53)

**Table 2:** Crude incidence rate ratios (bivariate models).^a^

Parameters		Crude incidence rate ratio		95% CI				*P* value	
					Upper		Lower		
HIV tweets 2016, person		1.006		0.975		1.043		.65	
Prevention 2016, person		1.082		1.003		1.183		.048	
Risk tweets 2016, person		0.976		0.931		1.024		.23	
HIV tweets 2016, institution		1.006		0.998		1.016		.13	
Prevention tweets 2016, institution		1.006		0.997		1.018		.16	
Risk tweets 2016, institution		1.155		0.876		1.651		.30	
HIV prevalence, 2015		1.002		1.001		1.002		<.001	
Social disadvantage index		1.104		1.075		1.134		<.001	
**Region of the United States**									
	Midwest		Ref^b^		Ref		Ref		Ref
	Northeast		1.286		0.985		1.683		<.001
	South		2.126		1.711		2.630		<.001
	West		0.967		0.749		1.250		>.99

^a^All tweet variables are reported in units of 100 tweets.

^b^Ref: reference.

### Multivariate Analyses

Multivariate models were used to test the adjusted effects for each of the 3 categories of tweets, for individuals and institutions separately, on HIV incidence in the following year. These models are summarized in Tables 3 and 4.

In all 6 models, HIV prevalence in 2017 was positively associated with HIV prevalence in 2015 and social disadvantage index. Additionally, all 6 models showed a significant difference in HIV incidence between the south and Midwest regions. Only one model, Model 1, showed statistically significant effect for a tweet variable on HIV incidence. In Model 1, HIV-specific tweets originating from individuals were negatively associated with HIV incidence at the county level in the following year, after adjusting for region, HIV prevalence, and social disadvantage index. Each additional 100 HIV-specific tweets per capita that originated from an individual in a given county was associated with a 3% decrease in the incidence rate of HIV in the following year, after adjusting for covariates.

**Table 3:** Multivariate models for tweets from individuals.^a^

		HIV incidence per capita, 2017					
		Model 1: HIV-specific tweets		Model 2: Prevention-specific tweets		Model 3: Risk-specific tweets	
Predictors		Incidence rate ratio (95% CI)	*P* value	Incidence rate ratio (95% CI)	*P* value	Incidence rate ratio (95% CI)	*P* value
HIV-specific Twitter activity, 2016		0.97 (0.94-1.00)	.04	N/A^b^	N/A	N/A	N/A
Prevention-specific Twitter activity, 2016		N/A	N/A	0.95 (0.90-1.01)	.13	N/A	N/A
Risk-specific Twitter activity, 2016		N/A	N/A	N/A	N/A	1.03 (0.86-1.24)	.73
HIV prevalence, 2015		1.00 (1.00-1.00)	<.001	1.00 (1.00-1.00)	<.001	1.00 (1.00-1.00)	<.001
Social disadvantage index		1.04 (1.02-1.06)	<.001	1.04 (1.02-1.06)	<.001	1.04 (1.02-1.06)	<.001
**Census region**							
	Midwest	Ref^c^	Ref	Ref	Ref	Ref	Ref
	North	0.90 (0.73-1.10)	.30	0.90 (0.74-1.10)	.29	0.90 (0.73-1.11)	.33
	South	1.40 (1.18-1.67)	<.001	1.41 (1.20-1.67)	<.001	1.41 (1.18-1.68)	<.001
	West	0.94 (0.78-1.15)	.55	0.95 (0.79-1.14)	.56	0.94 (0.77-1.14)	.51

^a^All tweet variables are reported in units of 100 tweets.

^b^N/A: not applicable.

^c^Ref: reference.

**Table 4:** Multivariate models for tweets from institutions.^a^

		HIV incidence per capita, 2017					
		Model 4: HIV-specific tweets		Model 5: Prevention-specific tweets		Model 6: Risk-specific tweets	
Predictors		Incidence rate ratios (95% CI)	*P* value	Incidence rate ratios (95% CI)	*P* value	Incidence rate ratios (95% CI)	*P* value
HIV-specific Twitter activity, 2016		1.00 (0.99-1.00)	.92	N/A^b^	N/A	N/A	N/A
Prevention-specific Twitter activity, 2016		N/A	N/A	1.00 (0.99-1.01)	.996	N/A	N/A
Risk-specific Twitter activity, 2016		N/A	N/A	N/A	N/A	1.03 (0.86-1.24)	.73
HIV prevalence, 2015		1.00 (1.00-1.00)	<.001	1.00 (1.00-1.00)	<.001	1.00 (1.00-1.00)	<.001
Social disadvantage index		1.04 (1.02-1.06)	<.001	1.04 (1.02-1.06)	<.001	1.04 (1.02-1.06)	<.001
**Census region**							
	Midwest	Ref^c^	Ref	Ref	Ref	Ref	Ref
	North	0.90 (0.73-1.11)	.34	0.90 (0.73-1.11)	.34	0.90 (0.73-1.11)	.33
	South	1.41 (1.18-1.68)	<.001	1.41 (1.18-1.68)	<.001	1.41 (1.18-1.68)	<.001
	West	0.94 (0.77-1.14)	.51	0.94 (0.77-1.14)	.51	0.94 (0.77-1.14)	.51

^a^All tweet variables are reported in units of 100 tweets.

^b^N/A: Not applicable.

^c^Ref: reference.

## Discussion

### Principal Findings

In this study, we analyzed the association between geolocated HIV-related tweets within the United States and the future incidence of HIV infection. HIV-specific tweets were more likely to emerge in those locations in the United States that had a high incidence of HIV. The number of HIV-specific tweets made by institution-associated accounts was higher than that of individual tweets. Interestingly, risk-related information in institution-associated tweets was lesser than that in tweets made by individual users. However, we did not observe significant associations between the number of HIV-specific tweets made by institutions and county-level HIV incidence. In contrast, increased numbers of HIV-specific tweets made by individual users were significantly associated with the decreased number of HIV cases in the following year at the county level, even when controlling for the geographic location. These findings suggest that the source of the tweet plays an important role, with individuals tweeting less about prevention, and these individual tweets showed a strong association with the future outcomes of HIV infections.

Geolocated conversations regarding HIV infections were negatively associated with county-level HIV incidence. These findings suggest that locations with few HIV-related Twitter posts and conversations by individuals may indicate those that require targeted interventions. Thus, counties with high incidence of HIV infections and few tweets may indicate an opportunity for increased investigation and potential intervention.

There are several possible reasons for our observation of low incidence of HIV infections in counties with large numbers of HIV-related tweets in the previous year. Increased numbers of HIV-related tweets at the county level could indicate increased community involvement, policy initiatives, and resource utilization in a given county [31]. Additionally, increased numbers of HIV-related tweets by individuals could reflect increased activities in addressing various determinants of HIV risks, including limited institutional support and reduced access to health care [32]. Although studies have sought to incorporate real-time analysis of Twitter data in association with localized HIV incidence, up-to-date HIV epidemiological data is limited or inaccessible to researchers. It is critical that public health professionals and computer scientists collaborate to develop novel approaches in analyzing Twitter data in accordance with the available HIV epidemiological data.

Our findings corroborate those of Ireland et al [15] but they are in contrast to those reported by Young et al [8] who found a positive association between HIV-related tweets and HIV prevalence. Although our study was similar in concept to that conducted by Young et al [8], we used a more specific definition of HIV-related tweets by excluding keywords that were less sensitive in our training sample (eg, “fuck”) and including a variety of slang terms that were compiled by our young researchers. Moreover, our study may have some slight differences from that of Young et al [8] because of the time period in our study—we may have identified a more recent phenomena in the prevalence of HIV infections. Two previous studies [14,15] analyzed the association between the HIV-related tweets and the corresponding epidemiological data in the same time period as considered in our study, whereas Young et al [8] analyzed the Twitter data with the epidemiological data of the previous year. The analyses in this study mirror those reported by Young et al [8] because we also analyzed the epidemiological data from the year after obtaining our specified frame of tweets in 2016.

Our findings suggest that discourses on HIV and risky sexual behavior on Twitter may serve as a signal of sexual health outcomes at the aggregate level. However, the low effect size and nonsignificant results of some of the models make it difficult to state this fact conclusively. It is clear that HIV-related discourse is geographically concentrated, and in coordination with epidemiological surveillance efforts, it may be used to inform intervention efforts. Despite the relative rarity of direct discussion of HIV on Twitter, this social media platform is still an important medium for conversations regarding HIV and health behaviors. Given its wide user base, Twitter can serve as a platform for discussing useful HIV prevention strategies, and such platforms deserve further investment as tools to end the HIV epidemic.

This study has several strengths. Our analysis combined NLP and manual coding, thereby allowing for coding of a large number of tweets for in-depth meaning, while preserving context. Our use of geolocated tweets allowed for location-based analysis with epidemiological and census data. These aspects of our study allowed for contextualized analysis of Twitter data, which may be useful for targeted interventions across the United States.

### Limitations

This study has several limitations. First, we did not evaluate model significance by using multiple correction comparisons. Our study only used a set of geolocated tweets from 2016, which greatly reduced the available sample. This analysis included 10.8% (339/3141) of all the counties and there may have been different Twitter discourses in other regions that were not included in this analysis. It is possible that tweets that were not geolocated in that year could have revealed additional information about the nature of HIV-specific tweets relative to HIV incidence. Second, we excluded Spanish tweets, which limited our ability to capture the web-based discourse among Latino men. Third, since we focused on deidentified Twitter data, our study does not contain information on individual characteristics or behaviors.

### Conclusion

With the increase of public discourse through Twitter, public health efforts leveraging this social medium are needed. Social media platforms such as Twitter offer an opportunity for health professionals to monitor population health and promote HIV disease prevention. We observed a negative association between HIV-specific tweets made by individual users and HIV incidence in the following calendar year at the county level. Our study underscores the importance of social media as a crucial aspect in the lives of individuals, as these discourses might unearth the youths’ knowledge, attitudes, and beliefs related to HIV. Public health efforts seeking to use social media as a tool for HIV surveillance and intervention are warranted.

## Abbreviations

IRR: incidence rate ratio
NLP: natural language processing
PrEP: pre-exposure prophylaxis
STI: sexually transmitted infection

## References

[1] Centers for Disease ControlPrevention (2017). https://www.cdc.gov/hiv/pdf/library/reports/surveillance/cdc-hiv-surveillance-report-2017-vol-29.pdf.

[2] Smith Aaron (2018). https://www.pewresearch.org/internet/2018/03/01/social-media-use-in-2018/.

[3] Perrin A, Anderson M (2019). Facttank: News by the Numbers.

[4] Cabrera-Nguyen EP, Cavazos-Rehg P, Krauss M, Bierut LJ, Moreno MA (2016). Young Adults' Exposure to Alcohol- and Marijuana-Related Content on Twitter. J Stud Alcohol Drugs.

[5] Cavazos-Rehg PA, Krauss M, Fisher SL, Salyer P, Grucza RA, Bierut LJ (2015). Twitter chatter about marijuana. J Adolesc Health.

[6] Gabarron E, Serrano JA, Wynn R, Lau AY (2014). Tweet content related to sexually transmitted diseases: no joking matter. J Med Internet Res.

[7] Kreniske P (2017). Developing a culture of commenting in a first-year seminar. Computers in Human Behavior.

[8] Young SD, Rivers C, Lewis B (2014). Methods of using real-time social media technologies for detection and remote monitoring of HIV outcomes. Prev Med.

[9] Broniatowski DA, Jamison AM, Qi S, AlKulaib L, Chen T, Benton A, Quinn SC, Dredze M (2018). Weaponized Health Communication: Twitter Bots and Russian Trolls Amplify the Vaccine Debate. Am J Public Health.

[10] Chou WS, Oh A, Klein WMP (2018). Addressing Health-Related Misinformation on Social Media. JAMA.

[11] Charles-Smith LE, Reynolds TL, Cameron MA, Conway M, Lau EHY, Olsen JM, Pavlin JA, Shigematsu M, Streichert LC, Suda KJ, Corley CD (2015). Using Social Media for Actionable Disease Surveillance and Outbreak Management: A Systematic Literature Review. PLoS One.

[12] Sinnenberg L, Buttenheim AM, Padrez K, Mancheno C, Ungar L, Merchant RM (2017). Twitter as a Tool for Health Research: A Systematic Review. Am J Public Health.

[13] Kalyanam J, Katsuki T, R G Lanckriet Gert, Mackey TK (2017). Exploring trends of nonmedical use of prescription drugs and polydrug abuse in the Twittersphere using unsupervised machine learning. Addict Behav.

[14] Ireland ME, Chen Q, Schwartz HA, Ungar LH, Albarracin D (2016). Action Tweets Linked to Reduced County-Level HIV Prevalence in the United States: Online Messages and Structural Determinants. AIDS Behav.

[15] Ireland ME, Schwartz HA, Chen Q, Ungar LH, Albarracín Dolores (2015). Future-oriented tweets predict lower county-level HIV prevalence in the United States. Health Psychol.

[16] Lazer D, Kennedy R, King G, Vespignani A (2014). Big data. The parable of Google Flu: traps in big data analysis. Science.

[17] Schwartz H, Eichstaedt J, Kern M, Dziurzynski Lukasz, Lucas Richard E., Agrawal Megha, Park Gregory J, Lakshmikanth Shrinidhi K., Jha Sneha, Seligman Martin E. P., Ungar Lyle (2013). Characterizing Geographic Variation in Well-Being Using Tweets. Proceedings of the 7th International AAAI Conference on Web and Social Media.

[18] Schwartz H, Giorgi S, Sap M, Crutchley P, Ungar L, Eichstaedt J (2017). DLATK: Differential language analysis ToolKit. Proceedings of the 2017 EMNLP System Demonstrations.

[19] Lui M, Baldwin T (2012). langid.py: an off-the-shelf language identification tool. Proceedings of the ACL 2012 System Demonstrations.

[20] Sap M, Park G, Eichstaedt J, Kern M, Stillwell D, Kosinski M, Ungar L, Schwartz HA (2014). Developing Age and Gender Predictive Lexica over Social Media. http://emnlp2014.org/papers/pdf/EMNLP2014121.pdf.

[21] Preoiuc-Pietro D, Ungar L (2018). User-Level Race and Ethnicity Predictors from Twitter Text. https://www.aclweb.org/anthology/C18-1130/.

[22] Young SD, Rivers C, Lewis B (2014). Methods of using real-time social media technologies for detection and remote monitoring of HIV outcomes. Prev Med.

[23] Centers for Disease ControlPrevention (2017). https://www.cdc.gov/nchhstp/atlas/index.htm.

[24] Hallett TB, Zaba B, Todd J, Lopman B, Mwita W, Biraro S, Gregson S, Boerma JT, ALPHA Network (2008). Estimating incidence from prevalence in generalised HIV epidemics: methods and validation. PLoS Med.

[25] Buot MG, Docena JP, Ratemo BK, Bittner MJ, Burlew JT, Nuritdinov AR, Robbins JR (2014). Beyond race and place: distal sociological determinants of HIV disparities. PLoS One.

[26] Bauermeister JA, Connochie D, Eaton L, Demers M, Stephenson R (2017). Geospatial Indicators of Space and Place: A Review of Multilevel Studies of HIV Prevention and Care Outcomes Among Young Men Who Have Sex With Men in the United States. J Sex Res.

[27] US Census Bureau, American Community Survey, 2016 American Community Survey 1-year estimates.

[28] Agresti A (2018). An introduction to categorical data analysis.

[29] R Foundation (2013). R: A language environment for statistical computing computer program.

[30] Venables W, Ripley B (2002). Modern Applied Statistics with S.

[31] Noguchi K, Handley IM, Albarracín Dolores (2011). Participating in politics resembles physical activity: general action patterns in international archives, United States archives, and experiments. Psychol Sci.

[32] Wallerstein N, Duran B (2010). Community-Based Participatory Research Contributions to Intervention Research: The Intersection of Science and Practice to Improve Health Equity. Am J Public Health.

